# Insecure Attachment and Other Help-Seeking Barriers among Women Depressed Postpartum

**DOI:** 10.3390/ijerph17113887

**Published:** 2020-05-30

**Authors:** Emily Cacciola, Elia Psouni

**Affiliations:** Department of Psychology, Lund University, P.O. Box 213, SE221-00 Lund, Sweden; emily.cacciola@gmail.com

**Keywords:** postpartum depression, postnatal depression, maternal attachment, help seeking, support seeking, attachment styles

## Abstract

When untreated, postpartum depression (PPD) can severely, negatively affect maternal health, child development, and the wellbeing and functioning of the entire family. Yet, despite screening and treatment programs for PPD, many women who experience depression with onset in the postpartum year do not communicate their symptoms. Negative relational experiences early in life, such as not receiving sensitive help and support when needed, often result in so-called insecure attachment styles, and there is evidence that these may contribute to the development and maintenance of PPD. However, the role of insecure attachment styles in non-help-seeking is unknown for this group. Using mixed quantitative and qualitative methodology, we identified help-seeking barriers of women who experienced depression with onset in the postpartum year but who had not sought help for their depression (N = 37), and explored links to their attachment orientations as assessed through both self-reported attachment style and narrative based attachment script assessment. The sample was non-normative regarding attachment, with an over-representation of avoidant attachment styles. Help-seeking barriers varied systematically with the mother’s adult attachment style. Specifically, convictions of a strong self and lack of trust in healthcare professionals constituted a common barrier among women with avoidant attachment styles, while unrealistic expectations about motherhood constituted a barrier for women with secure attachment styles. This new knowledge on how barriers to communicating symptoms and seeking help when suffering from PPD vary systematically with attachment orientation can help formulate individualized, and therefore more efficient, approaches to addressing non-help-seeking behavior in women who suffer in silence.

## 1. Introduction

While mild depressive symptoms that typically decline after four weeks postpartum are common among women after giving birth [1], for many women these feelings are not transient [2,3,4]. Indeed, the Diagnostic and Statistical Manual of Mental Disorders (DSM-5 [5]) defines postpartum depression (PPD) as a major depressive disorder (MDD) with onset in the peri-partum, which includes the period during pregnancy and the four weeks following delivery [5]. However, as it is well-established that women may develop depressive symptoms, and continue to suffer for up to one year postpartum and still remain undiagnosed [6], many researchers and clinicians have argued for, and used, a wider onset specifier (e.g., [7]). Thus, an onset extended to one year postpartum is currently used in most research on maternal perinatal health [8]. PPD is recognized as a worldwide mental health problem negatively affecting maternal health [6], child development [9,10], and family functioning [11,12]. Importantly, while disclosure of the distressing symptoms within the healthcare context, here referred to as “help-seeking” [13], often leads to professional help and subsequent relief, not receiving help for PPD has been shown to sustain the condition [14]. 

However, access to help is dependent on women seeking it [15]. Although screening for symptoms of depression in mothers is included in the perinatal healthcare practices of many countries, there is alarming evidence that many women do not disclose their symptoms [3,16,17]. Different possible barriers to seeking help have been discussed in the literature, including fear of stigma of mental disease [3,18,19], fear of losing parental rights [17], and insufficient knowledge about PPD and related treatment possibilities [3,20]. Guilt and shame, stemming from feelings of not knowing how to care for a child, or feelings that the mothering role is not fulfilling above all, may also constitute a help-seeking barrier. This has been shown to be the case for mothers in general [21,22], and when suffering from PPD in specific [3,6,14,23,24,25].

### 1.1. Seeking Help and Attachment Style

Attachment theory [26,27] provides a theoretical framework for understanding individual socioemotional development within the context of early parent–child relationships, and suggests that different patterns of experiences in these relationships are generalized, through development, into relatively stable mental models and individual attachment styles [28]. Individual attachment styles in adulthood are likely to influence willingness to disclose mental health problems [29]. The secure attachment mental models that often emerge from early childhood experiences with available, sensitive, and responsive caregivers, include a positive view of self and a sense that other people are trustworthy and will be helpful in times of distress [26,27]. Thus, when faced with distressing difficulties, individuals with secure attachment styles tend to turn to others for help. The anxious attachment mental models associated with inconsistent experiences of caregiver availability and support involve positive views of others but negative views of self [28]. Fueled by high levels of relational anxiety and fear of being abandoned [30], individuals with anxious attachment styles tend to magnify distress signals in an effort to secure relational proximity. For this reason, they are also likely to disclose their distress to professionals [31].

By contrast, individuals with avoidant attachment styles are less likely to seek help and support compared to individuals with anxious attachment style [31,32]. The avoidant attachment mental models that often emerge from repeated experiences of unresponsive or rejecting caregivers during early childhood comprise negative views of others combined with positive views of self [33]. Thus, individuals with avoidant attachment styles are more unlikely to trust others [30] or even acknowledge that they are in need of help. Ignoring potential support possibilities and turning instead to self-soothing is common [28]. Furthermore, when faced with emotional distress, these individuals often use deactivating strategies, such as conscious and unconscious affective disengagement, and dismiss their negative emotions and difficult experiences. This may in turn cause healthcare professionals to withdraw, as no apparent help seeking is displayed [8]. Finally, individuals with disorganized attachment hold negative models of both others and themselves, and appear torn between needing relational closeness when in distress while simultaneously distrusting other people [34]. 

Attachment styles in adulthood are also likely to influence an individual’s vulnerability for mental health problems. Specifically concerning depression, attachment anxiety has been linked to depression in general [27,35], increased depressive symptoms in fathers in the perinatal period [36], and increased risk of postpartum depression in mothers [34,37,38,39,40]. Attachment avoidance has also been associated with increased depressive symptoms in women postpartum [41]. In fact, meta-analytical data reveal an overrepresentation of both anxious and avoidant adult attachment mental models in a sample of depressed mothers, compared to that of non-clinical samples [42]. However, most research on maternal PPD has focused on women who either were referred to, or themselves sought, professional help for their condition [14,43]. It is entirely unknown whether features of insecure attachment, avoidant or anxious, constitute help-seeking barriers among women who also experience depression with onset in the postpartum year. 

### 1.2. The Present Study

In the present study, we aimed to assess if, and how, attachment insecurity in adulthood contributes to non-help-seeking among women who experience depression with onset in the postpartum year. Attachment insecurity was operationalized as attachment avoidance, attachment anxiety, or attachment disorganization, as assessed through a combination of a self-report measure of attachment style, and an assessment of the mother’s attachment scripts. Attachment scripts are sensorimotor-affective schemas of event sequences, which developed during infancy and early childhood [44] and encapsulate information about usual caregiver behavior in different situations. Consistent and coherent support from caregivers during infancy and early childhood is likely to result in readily accessible scripts of interactions, in times of need, with significant others (parents, partners, close friends) who are willing and able to provide support and soothing. By contrast, inconsistent or ineffective caregiver support in times of distress will likely result in less accessible or incomplete scripts, or scripts that include negative expectations about significant others [45]. Non-help-seeking was operationalized as non-disclosure, towards healthcare professional (midwifes, nurses, physicians, psychologists, or other mental health therapists), of one’s symptoms indicative of suffering and poor mental health. We therefore considered both the act of not seeking contact with a healthcare professional and the act of not disclosing symptoms when in contact with healthcare professionals. Finally, we operationalized PPD as a combination of both a high level of self-reported depressive symptoms and the outcome of a clinical diagnostic interview.

Based on previous suggestions and findings concerning help-seeking behavior in general (e.g., [29,30,33]), we expected weak attachment scripts and an overrepresentation of women with avoidant attachment styles among non-help-seeking women who are depressed in the postpartum. A second aim was to identify the broad spectrum of help-seeking barriers operating among women who experience depression with onset in the postpartum year but who nevertheless do not disclose their symptoms, and examine the extent to which the specific reasons for each woman’s non-help-seeking may differ systematically with attachment style. 

## 2. Materials and Methods

### 2.1. Design and Setting

We conducted a study based on a combination of quantitative and qualitative methods, assessing depression and attachment quantitatively, through self-report questionnaires and narrative-based techniques, and thoughts and feelings related to help seeking qualitatively, through in-depth, semi-structured interviews. This design could elicit rich descriptions of mothers’ experiences of depression and help seeking, while allowing assessment of attachment styles and severity of depressive symptoms based on quantitative measures whose psychometric qualities are well established. The study was conducted in Sweden, a Nordic country with a lifetime incidence of depression among women of about 36% [46], with higher levels of depression in winter when sunlight is reduced [47] and 8–15% prevalence of maternal PPD [48]. 

### 2.2. Procedure

The study was announced in social media platforms where women share birth giving and early parenting experiences. As the potential research participant may not, for different reasons, identify herself as specifically suffering from depression, the announcement did not explicitly include this term. Instead, women were invited to participate if they (a) were at least 18 years’ old; (b) had within the past 24 months given birth to a healthy, full-term baby; (c) perceived themselves as feeling mentally unwell in the postpartum period; and (d) had not indicated this to the healthcare services or otherwise sought professional help for their depression. While a period of one year post-partum is commonly used in research focusing on health and wellbeing the perinatal period (e.g., [3,40,43]), we employed in the announcement a widened timeframe of 24 months postpartum, in line with other studies concerning barriers to help-seeking (e.g., [18,25]). We did this in order to include mothers who developed depressive symptoms towards the end of the first year, as well as those who did not identify themselves as feeling mentally unwell until a long time after onset. This widened timeframe also takes into consideration the high likelihood that many potential participants could have been suffering a prolonged depression since they would not have received professional treatment. 

**Figure 1:**
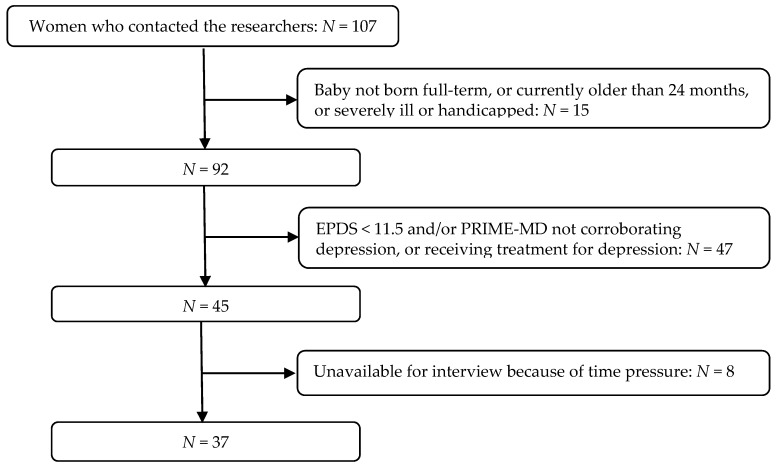
Recruitment procedure steps and total sample. Note: EPDS: Edinburg Postpartum Depression Scale; PRIME-MD: Primary Care Evaluation of Mental Disorders, see *Measures,* below.

In total, 107 women contacted the researchers within a period of two weeks. For 15 of those it was revealed in this initial contact that their babies were not born full-term, were older than 24 months, or suffered severe illness/handicap. The remaining 92 women were invited to participate by filling in in the digitalized self-report part of the study on-line, at a place and time of their own choice. For 45 women, their depression was corroborated by the study depression measures (see below) and it was also corroborated that they had not received and were not currently receiving treatment for depression. These women were asked to participate with an interview. In total, 37 interviews were carried out. The eight women who were not interviewed reported lack of time as their reason for dropping out (see Figure 1). Interviews were carried out at a designated room at the university campus. They were conducted by the authors, both females and licensed clinical psychologists. On average, interviews lasted about 45 min. 

As deemed by a Research Ethics Committee at the Department of Psychology, Lund University, all procedures in the study were in accordance with the ethical guidelines for human subjects’ research determined by Swedish law. Contact was established by initiative of potential participants. Participation was strictly voluntary. Informed consent was obtained from all individual participants. Participants were informed that they could at any point end their participation (no questions asked) and were encouraged to contact the researchers, at any time during or after participation, should any questions or concerns arise. Arrangements had been made for quick referrals in such an event. The information gathered was private and sensitive but was collected anonymously, as participants only identified themselves with a code known only to themselves. No code lists were constructed. Any identity cues in the interviews were removed upon transcription. At the end of the interview, all participants were informed of ways of establishing contacts with clinical psychologists within or outside the national healthcare services.

### 2.3. Measures

#### 2.3.1. Depression

As a first indicator of PPD, mothers filled in the Edinburgh Postnatal Depression Scale ((EPDS): [49]; validated Swedish version: [50,51]), a 10-item self-report with documented validity and reliability [2,52]. In cases of suspected depression, as indicated by an EPDS cut-off score ≥ 11.5 [51], a telephone interview was carried out by a clinical psychologist (1st author), using the depression module of the Primary Care Evaluation of Mental Disorders [53], a clinical instrument designed for the diagnosis of depression.

#### 2.3.2. Attachment Style

The attachment style questionnaire ((ASQ): [54]; Swedish ASQ-Sw: [55]) is a 40-item, self-report measure of attachment. Items (e.g., “I feel confident that other people will be there for me when I need them”) scored on 6-point Likert scales inform the subscales, “Confidence” (indicating attachment security), “Need for Approval” and “Preoccupation with Relationships” (indicating attachment anxiety), and “Discomfort with Closeness” and “Relationships as Secondary” (indicating attachment avoidance). Based on subscale score profiles and normative data [55], respondents are classified into the attachment styles of Secure, Anxious, Avoidant, or Disorganized. 

Attachment script assessment ((ASA): [56]; Swedish translation: [57]) is a validated narrative-based method assessing implicit schemas of interactions with attachment figures, signifying the degree to which the individual has comprehensive and accessible scripted, secure-base/safe-haven knowledge. Participants narrate stories, guided by prompt words that provide an outline indicating who is involved and what happens [45,58]. All four story titles addressing attachment were used, two concerning parent–child interactions (“Baby’s Morning” and “Doctor’s Office”), and two concerning interaction between romantic partners (“Camping Trip” and “Accident”). Transcribed stories were scored on 7-point scales. To receive a high score, a story must contain descriptions of the protagonists’ interactions including emotional communication and support, warmth, availability, and sensitivity [58]. Thus, a score of “7” indicates comprehensive attachment scripts that clearly demonstrate secure base/safe haven knowledge, while a score of “1” indicates apparent lack of secure scripts. A score of “4” is given to stories that contain sufficient, if rudimentary, evidence of a secure script [45,58]. Two example narratives appear in Table 1. 

#### 2.3.3. Help-Seeking and Help-Seeking Barriers

Participating women were interviewed regarding their behaviors, thoughts, and feelings about disclosing their symptoms. Interviews were conducted based on an interview guide developed for the purposes of the present study, inspired by [59,60], and available (in Swedish) upon request. The interview comprised fifteen open questions. These included some introductory questions in order to ease in the participant into the discussion, followed by questions covering the domains of Experiencing (e.g., “Have you told someone about what you are experiencing?”), Sharing (e.g., “Does something make it difficult for you to share with someone about what you are experiencing?”), Understanding (“Do you think there is something in you that makes you less likely to share these experiences?”), and Solving (e.g., “Do you think there is something that healthcare professionals could do or say in order to facilitate this sharing?”). Interviews lasted about 45 min. 

### 2.4. Material Preparation and Analysis

ASQ-subscale internal consistency ranged in the present study between 0.80 and 0.94. Based on subscale score profiles and normative data [55], the two authors independently classified mothers into the attachment styles of Secure, Anxious, Avoidant, or Disorganized. Inter-coder classification agreement was 0.89 (*χ*^2^ = 85.6, *p* < 0.0001). The two authors also scored, independently, participants’ ASA stories (the second author is a senior reliable coder, trained by the method developer). Stories were scored by story-title, without any link to the participant who narrated them. Inter-coder correlations were high (Camping Trip α = 0.97, Accident α = 0.96, Doctor’s Office α = 0.91, and Baby’s Morning α = 0.95). Internal consistency for the four stories was α = 0.68, somewhat lower than previously reported (e.g., [57]). Two sub-scores per participant were calculated, one averaging the two parent–child stories and one averaging the two stories concerning interactions between adult partners. A total ASA-score was also calculated. Using the score of “4” as cut-off (see [59]), ASA data were also dichotomized to denote evidence, or not, of secure attachment scripts in the participants’ ASA stories.

Interview transcripts were coded by the two authors, using directed content analysis. This is an analytical method that is well suited for identifying important aspects of the material without the use of preconceived categories [61], but with a recognition that an existing empirical and conceptual framework underlies the analytic process [62], and a dedication to further specify this conceptual framework. Based on previous reviews summarizing potential common help-seeking barriers among women who are depressed in the postpartum period, social myths and expectations and lack of knowledge, e.g., [3,23], as well as fear of losing parental rights [17], were considered as relevant themes, and therefore initial, generic coding categories. A general descriptor of inability or reluctance to disclose symptoms is also often encountered in the previous literature, e.g., [3,8] and was thus considered as another theme in the conceptual framework. Importantly, the attachment theoretical framework was summoned as potentially useful for specifying further this theme, through identifying reasons behind this inability or reluctance to disclose symptoms.

The goal of the analysis was to capture all themes in the transcribed material, categorize them, and label the categories directly from the text, working as closely with the material as possible. Following recommendations [62], the authors first read and re-read the verbatim transcribed material. The first author subsequently highlighted the full body of the material according to a first glance theme-representation, and then systematically coded [61], adding notes and thoughts in the text margins. In a third step, the coder named and defined preliminary themes, directly from the content. Next, the second author applied those themes, coding the entire body of the material, noting discrepancies and redundancy in the text margins. Initial agreement between coders was high (90%). Disagreements of excerpt coding were resolved in conference between the coders. The outcome of the analytical process was five themes, which were labelled jointly and directly from the content. 

**Figure 2:**
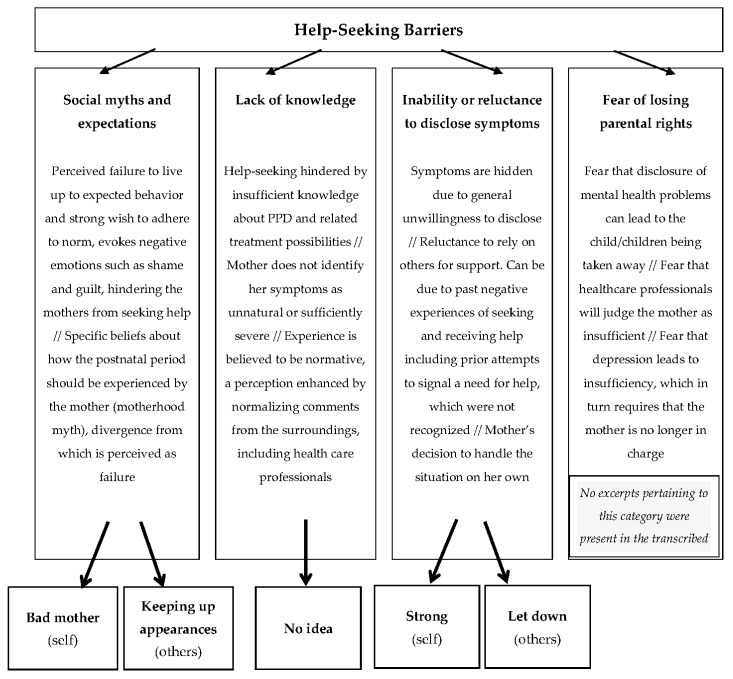
Links between the generic coding categories and the final categories.

Thus, our initial coding was deductive, guided by a set of predefined, generic themes concerning individual help-seeking barriers among women suffering with PPD, in accordance with [62]. However, in an effort to better understand these barriers and hopefully generate knowledge that may help identify ways to surpass or work around them, our directed content analysis did not only seek to verify the preexisting conceptual framework but also to further specify reasons behind individual inability or reluctance to disclose, working inductively from the participants’ interviews. We regarded this combination of deductive and inductive processes in our directed content analysis as necessary, see also [63,64]. Figure 2 illustrates the links between the predefined, generic coding categories/sub-categories, and the final categories, following recommendations by [65].

Mothers’ attachment styles were characterized based on the ASQ and ASA. These characterizations were then used for identifying whether the different help-seeking barrier themes, recovered from the directed content analysis, were present to different degrees in transcripts of mothers with different adult attachment styles. 

## 3. Results

### 3.1. Description of Participating Mothers

Participating mothers (*N* = 37) were between 27 and 43 years old (*M* = 32.5 years, *SD* = 4.4). Several (84%) had an education corresponding to at least three years of post-high-school full-time study (see Table 2, below). Five women (14%) had had assisted reproductive technology and five (14%) had had caesarian section deliveries. Sixteen participants (43%) reported having attended a discussion with a nurse at a childcare center, as part of a screening routine for postpartum depression. Those, and an additional eight mothers (*n* = 24, 65%) reported having been asked to fill in a depression self-rating questionnaire on one previous occasion. None of those mothers had explicitly sought help or been referred for further assessment or support.

Internal consistency for the EPDS in the present sample was α = 0.75. Mothers had a mean EPDS depression score of 19.7 (SD = 4.53). This is markedly high, compared to EPDS cut-off scores that denote suspected depression [2,25,51]. Furthermore, fifteen mothers (41%) reported recurring thoughts of harming themselves. Twenty-one mothers (57%) reported previous mental health problems: depressive episodes (n = 16, 43%), severe stress (n = 3, 8%), or a crisis reaction (n = 2, 6%). Notably, there was no association between EPDS scores and previous mental health problems, or any other demographic variables. 

### 3.2. Attachment Scripts and Attachment Styles in Non-Help-Seeking Mothers with PPD

According to ASA, 60% of mothers lacked secure attachment scripts. Based on the ASQ, 40.5% of mothers (*n* = 15) were classified as secure, 27% (*n* = 10) as avoidant, 24% (*n* = 9) as anxious, and 8% (*n* = 3) as disorganized. Mean ASQ-subscale scores indicating avoidance (“Discomfort with Closeness” and “Relationships as Secondary”) were overall high compared to reference values from normative data [54]. Cross-tabulation of ASQ-classifications and dichotomized ASA-scores indicated convergence of the two measures (*χ*^2^ = 13.51, *p* < 0.0001, *kappa* = 0.60), revealing that most mothers classified as having avoidant, anxious, and disorganized adult attachment styles (together comprising 60% of participants), also lacked secure attachment scripts. This was also corroborated by the systematic differences in mean ASA-scores depending on adult attachment style as determined by the ASQ (Table 3). Importantly, EPDS (depression) scores were neither related to ASQ-subscale nor ASA scores.

### 3.3. Help-Seeking Barriers among Mothers with PPD and Their Links to Adult Attachment Styles

Five themes emerged from the directed content analysis of the interviews: (i) strong, (ii) keeping up appearances, (iii) let down, (iv) bad mother, and (v) no idea. When referring to quantities, “few”, “about half”, and “many” was used to represent frequencies around 20%, 50%, and 80%, respectively.

#### 3.3.1. Strong

Extracts pertaining to this theme were present in many participants’ interviews, mainly of those mothers classified as having avoidant or secure attachment styles. An expectation that mothers should be able to endure any amounts of discomfort and handle things without needing or receiving help characterizes all extracts. There seems to be a perceived strength in not needing others in order to work through difficulties. The opposite, to ask for help and support, is experienced as a failure. As such, not displaying weakness is highly valued. Strength and a sense of autonomy are described as part of the woman’s character. As is staying in control and handling things on one’s own. Asking for help displays frailty and inability to live up to fundamentals of motherhood. Illustrative excerpts can be seen below:

“I am a person who likes to do things myself, I am strong. I perceive myself as strong and capable. That I have things under control... so yes, that is definitely part of it.”

“I feel... if I can care for my child and still manage to stay alive then it’s not that big of a problem that I should seek help for it. That is how I think. Handle things yourself. I am supposed to handle things myself, anyways.”

“I do not even visit the doctor when I am ill, physically ill. I don’t like to feel weak... If I fail to do something I work at it until I succeed.”

#### 3.3.2. Keeping Up Appearances

About half of participants expressed a strong drive to keep up appearances; not showing others how things really are. By making things look blissful and prosperous, though they are not, the women avoid having to break the mirage of thriving motherhood-as-expected. In answers pertaining to this theme, the women describe processes of hiding their real state so as not to let other people down. They convey that this is necessary since they failed to live up to the positive image of themselves, one that has been establish by themselves and others. Being a “good girl”, not causing trouble or discomfort to others, and taking responsibility for others’ well-being, also during the postpartum period, was presented as both desired for, and expected of, oneself. In those accounts of the postpartum period there is a clear declaration of no space for fatigue or sadness. Excerpts categorized in this theme were equally frequent in transcripts from women whose attachment styles were characterized as avoidant or anxious. Typical excerpts, illustrative of this theme, are:

“I’m your typical “good-girl”, you know, making sure everyone has a good time. It’s always important that it’s nice and happy. Yes, I think it’s because I’m always supposed to... everything must look so successful.”

“I have people in my life who adore me, put me on a pedestal, are very proud of me. Somewhere maybe I am afraid to... I have an image of the way I am supposed to be. And to let someone in and admit I need help; I would inevitably disappoint someone.... I think I am trying to live up to an image of what I am supposed to be like.”

“I think I am very good at keeping up appearances towards other people... I didn’t think I would feel this bad and it’s hard for me to say how awful I feel because I start comparing myself to others who, I figure, felt even worse.”

#### 3.3.3. Let Down

About half of participating mothers expressed experiences of being let down or mistreated by healthcare professionals, and consequently lacked trust in the healthcare system. These women describe an awareness of their suffering and a wanting for help, expressed as if they would have accepted help if offered, yet their experience with healthcare professionals prompted them to withdraw. Excerpts classified under this theme were mostly encountered in the interview transcripts of women classified as anxious or disorganized, but the theme was also present in the narratives of five mothers with secure attachment styles. Some mothers describe being met with attempts to normalize their experience, which would cause them to retract any further attempts of talking about their mental state. Some of these women reported having signaled need for help, which was missed or ignored. Follow-up probes could not clarify what the signal was, or how salient, yet participants insisted that someone should have sensed or seen their suffering. Some typical excerpts are:

“I send signals to healthcare professionals. But they miss my signals. In hindsight this has made me very disappointed. Can’t see myself how I feel, so I need someone else to see me, but they don’t.”

“The answer “everyone feels this way, it will pass” is not an okay answer when you feel like shit. You shouldn’t have to yell, cry or show apathy just so that the healthcare system will understand you are feeling bad.”

“I am let down, left alone, completely traumatized. The reason I have not gotten any help is incompetence in healthcare. They should have seen something wasn’t right.”

#### 3.3.4. Bad Mother

This theme was expressed by few mothers, characterized mainly by secure attachment. It reflects a strong concern about being a “bad mother” because of not knowing how to instinctively love her child and enjoy parenting. Shame and guilt from not being able to fulfill own and external, unrealistic expectations of motherhood, and over not feeling the expected feelings, is expressed. The discrepancy between reality and the idealized image of motherhood is described to cause the women to withdraw from any possible support, as the contrast makes it harder to talk about the actual experience without perceiving oneself as a bad version of a mother. Illustrative for the theme excerpts are:

“Somewhere deep inside there is a shame of some kind. I mean, this makes me a really bad mother. If you can’t give your baby what it needs, how do you feel? You feel completely worthless. I feel completely worthless as a human being.”

“I am ashamed. Shame is the main reason. The fact that I longed so for my baby, I was a painfully happy pregnant person, I just reveled in it all. The bed was ready, everything was prepared and I just savored it. So naturally I am ashamed, I don’t want to admit that things aren’t the way I had imagined.”

“I mean, if someone asks, “How are you?” when you have a little baby, and I say, “It’s completely horrible, it sucks.” I would flinch... I really would. Now is the time to be happy, I am supposed to be happy that I have a baby. That is not the time to tell how hard it is, how terrible you feel or that you changed your mind.”

#### 3.3.5. No Idea

This theme was expressed by few mothers characterized mainly by disorganized attachment. Narratives convey profound ignorance regarding what to expect from parenting, a sense of not knowing how to care for the child or what to expect. These accounts also convey ignorance regarding mental illness; the women seemingly unaware of what can constitute depressive symptoms. With limited insight into what to expect in terms of taking care of an infant in the postpartum period, the depressive symptoms are described as being interpreted as a general dissatisfaction with parenthood. Comments from the surrounding environment appear to have served to further normalize the depressive symptoms, causing the suffering women to withdraw.

“I never understood what was wrong with me, until now; I thought my sadness meant I didn’t want to be a mother. I didn’t realize how bad I was feeling. This is what it’s like to be a parent everyone around me kept telling me, and I didn’t know, you’re not supposed to feel that horrible.”

“Well... It’s hard to seek help when you don’t realize you have a problem. I had no idea what it’s like to have a baby, so there’s nothing to... you know? It’s really hard, I think that if I had understood that I could be helped I would have spoken. But I didn’t understand, and everyone was telling me it was normal, so I believed it.”

“I had no idea, it’s incredible. And I have no idea why I couldn’t grasp that it’s not okay to feel this way. I don’t think I ever understood how bad I felt, how bad it really was.”

## 4. Discussion

Previous research concerning the role of the mother’s mental models of attachment in the occurrence of maternal postpartum depression has been fundamentally based on women who either were referred to, or themselves sought, professional help for their condition [14,43]. Instead, based on a sample of non-help-seeking women suffering from PPD, the present study findings demonstrate, for the first time, systematic links between the participants’ adult attachment styles and their reasons for not seeking help. Confirming our hypothesis, there was also an over-representation of avoidant attachment styles among non-help-seeking mothers with PPD. Somewhat surprising, we also found that many non-help-seeking mothers with PPD had secure adult attachment styles. 

### 4.1. Attachment Styles and Non-Help-Seeking

Overall, the sample was non-normative with respect to attachment, with higher frequency of women with insecure attachment styles and lacking secure base scripts, compared to other clinical [42] and non-clinical [42,66] groups. This is consistent with our hypothesis and previous findings on women suffering from PPD [34], and research with other non-help-seeking populations [32]. The larger proportion of disorganized attachment styles in the sample, compared to non-clinical groups, is in line with previous research confirming the increased vulnerability of individuals with disorganized attachment style to suffer from PPD [32,37,39,40]. Our findings also hint that these individuals may not necessarily disclose their symptoms when suffering from PPD. 

Previous research based on women with referrals has linked anxious attachment to elevated risk for PPD [37,39,40]. While the inclination of anxiously attached individuals to use hyper-activating strategies generally promotes making contact with healthcare professionals, our findings reveal that anxious attachment is as frequent among non-help-seeking women. Indeed, PPD may also augment the more fearful features in anxiously attached women, intensify their relational anxiety [32,33], and lead them to refrain from sharing their thoughts and feelings, out of fear of rejection. This is also consistent with our finding that a frequently operating help-seeking barrier among those women was a fear of letting other people down, or of receiving negative judgement, in case they would disclose their suffering. This fear may even have prevented some anxiously attached mothers from participating in the present study. 

Consistent with a reluctance to communicate mental distress associated with attachment avoidance, a large proportion of women with avoidant attachment styles was present among the non-help-seeking group, compared to groups of women who receive professional help for PPD (e.g., [39]). Furthermore, moving beyond categorical classifications of attachment, a high degree of avoidant strategies were present among the majority of women in our sample. These findings should not be surprising. Attachment avoidance has been associated with a general tendency to under-report distressing symptoms in difficult situations associated with parenting (e.g., [67]). Avoidant strategies were clearly associated with (over)valuing self-sufficiency and self-composure and, therefore, disinclination to communicate distress, within but also outside the healthcare system. The aspiration to convey capability, control, and deny any instances of uncontained emotion, was the most salient barrier to help-seeking among these women. 

Individuals with secure attachment are both less likely to suffer from mental health problems [68] and more likely to seek help when suffering from mental health problems [31]. Thus, the relatively large proportion of mothers with secure attachment styles in the present non-help-seeking sample was surprising. Our findings suggest that the strain of battling depression in the postpartum period may cause otherwise well-functioning interpersonal patterns to falter. More importantly, our findings indicate that unrealistic expectations about motherhood, such as the idea that women should immediately and instinctively know how to care for their children, may be both a stressor [21,22] and a help-seeking barrier. Such expectations about motherhood were common help-seeking barriers among women with secure attachment styles. Apparently, despite the fact that these women held attachment scripts that clearly demonstrated knowledge of secure base/safe haven interactions, this did not prevent or protect them. Overwhelmed by strong feelings of shame and self-failure from not experiencing the joy and fulfillment they expected and were expected to feel, they were blocked from disclosing their suffering and from seeking help for their PPD. Thus, secure attachment does not necessarily constitute a protective barrier in coping with such expectations, which in fact appear to override the healthy help-seeking attitude characteristic of secure attachment. 

### 4.2. Demographic Characteristics and Non-Help-Seeking

Some findings concerning demographics deserve further comment. First, supporting previous findings that psychiatric illness prenatally may constitute a risk for future mental illness (e.g., [4,50]), several women in the present sample reported previous episodes of depression. As recent studies have indicated that the negative outcomes of maternal PPD on child development are more likely in the presence of a comorbid psychiatric disorder [69], further research is warranted to explore specific patterns of PPD and comorbid psychiatric illness among non-help-seeking women. 

Second, there was an overrepresentation in the present sample of mothers who had used assisted reproductive technology. Actual experiences may significantly diverge from prenatal expectations for these women, resulting in feelings of shame and guilt that stops them from expressing their depressive symptoms postpartum [43,70,71]. 

Third, several women had filled in the EPDS on a previous occasion. It is reasonable to assume that many did not share their true feelings when doing so, as they did not receive a referral. Alternatively, those women’s depression may have expressed itself through symptoms not addressed by the EPDS [36,72]. Close and continuous contact with the healthcare professional may thus be essential for counteracting the reasons that lead many women to understate their symptoms also in self-report measures such as the EPDS. 

### 4.3. Limitations

The study relied on the EPDS as an initial, screening indicator of depression, in line with other research and clinical praxis [1,2,20,73]. However, the validity of the EPDS, especially outside of the initial postpartum period that it was originally developed for, is not established beyond doubt [2,25,48,73]. Since the role of the EPDS in the present study procedures was merely for screening, the relevant issue here is the risk of false negatives—women suffering from depression whose self-reported symptoms through the EPDS did not qualify them for a follow-up clinical interview. To limit this risk, we employed a generous cut-off score, but it cannot be excluded that some depressed women were missed. Notably, participants in the present study reported high levels of depressive symptoms, which may also explain the lack of association between previous history of depression and the women’s EPDS scores. While previous mental illness is associated with increased risk of suffering PPD, there is no evidence that the severity of depressive symptoms will be higher among women with previous history of mental illness. 

Symptoms of depression generally involve a negative self-image [74]. Since the ASQ includes items addressing self-image, the respondent’s depressive state may have confounded ASQ-scores and related attachment style classifications. To limit this risk, we used two instruments to capture both a more implicit facet of the attachment representation with the ASA [45,57], and a more explicit facet as determined by the ASQ. The two instruments converged, but it cannot be excluded that the depressed mothers’ ASA narratives were also influenced by their negative mood.

The number of participants in the resent study is entirely justified by the qualitative methodology employed, yet it is limited, and geographically restricted. This limits the transferability of our findings to other settings and cultural contexts. Thus, future research ought to investigate whether the present findings replicate in cultural contexts where the values and social expectations around mothering may differ. Given difficulties in recruiting women suffering from PPD for research (e.g., [75]), and the additional difficulty when targeting non-help-seeking women, the present study relied on social media for reaching to potential participants with information, and offered Skype interviews for data collection, which potentially further limits the study generalizability. On the other hand, these means of reaching parents in the postnatal period may deserve consideration in both research and practices for providing support for PPD, as many women in the postpartum period report that merely leaving the home is a struggle [76]). Reaching to those burdened by depressive symptoms through the use of mobile techniques may be particularly relevant for non-help-seeking populations.

Finally, the present study focused solely on women, despite increasing evidence of worrying prevalence of postnatal depression also among fathers [69,77,78]. Considering also the evidence of mutual influences, between co-parents, regarding wellbeing and mental health in the postnatal period [36], but also parenting practices [57], it is important that future studies expand their focus to include fathers. 

## 5. Conclusions

It may seem odd that the women suffering with PPD in the present study are referred to as non-help-seekers. However, reaching out in order to participate in the study cannot be labeled help-seeking behavior, since the explicit reason for this contact was not to receive professional help. Importantly, participation was anonymous and involved no commitment to any further contact with the researchers or healthcare professionals. Study participation, however, reflects a certain degree of transparency and willingness to share their experience. The fact that they were prepared to participate in the present study indicates that these women’s help-seeking barriers are relative, rather than absolute. It may well be the case that the women least inclined to seek professional help are perhaps equally disinclined to talk about their experience in a research setting, effectively entirely falling off the scientific radar.

The present study highlights different reasons behind women suffering from PPD ultimately not seeking the professional help they need. As unrealistic expectations of motherhood are shown to play a major role as help-seeking barriers for women with a secure attachment style, it is clear that not every aspect of non-help-seeking in PPD is related to attachment insecurity. For the large group of women suffering from depressive episodes postpartum, the contrast between actual experiences and expectations may result in keeping their suffering hidden from healthcare professional, friends, family, and partners. While many participants expressed having deliberately hidden their true psychological state, some, in particular those with high preoccupied or disorganized attachment features, spoke of having tried to convey their need for help and having experienced rejection, which in turn caused them to withdraw. Evidently, the help-seeker and the help-provider may have different ideas of what constitutes “asking for help”. Appearing overtired may for some mothers constitute a signal of needing help, one that the healthcare professional may dismiss as most mothers of babies are tired. Similarly, appearing composed and in control may also increase the risk of suffering unnoticed. Thus, descriptions of what constitutes help seeking ought to be further scrutinized. Furthermore, increased awareness, among healthcare professionals, of how attachment dispositions influence help-seeking behaviors appears crucial. Further insights to this end will likely be essential for detecting PPD. In particular, future interventions based on this knowledge may prove especially helpful for the large group of mothers with insecure attachment styles, as the results of the present and other studies show that mothers with insecure attachment mental models are both more likely to suffer from depression and less likely to seek help.

## Figures and Tables

**Table 1:** Example attachment script assessment (ASA) narratives (“Doctor’s office”), with and without secure base scripts.

***Story 1, Narrative with no Evidence of an Attachment Script***
This is about **Tommy**, a boy who may be five years old. He is out cycling, he got a **bike** for his birthday and he is very happy. And he cycles a little fast and **hurries**, and hits on a rock so he rolled, overturned with the bike and then he hit his arm a lot so that it **hurt** really bad. Some people came by who saw him and helped him home and they had to go to the **doctor** and show this arm that was hurt. He was very sad and **cried** a lot. When they came in to the doctor, she looked at his arm and felt it and there was a deep wound so she got a syringe and gave him a **shot** so that it would not become infected. He was not damaged otherwise, everything looked ok, they did not need to X-ray. And then when they left they went past a **toy** store and bought a toy because it had been a little boring for the boy, and then they went home.
***Story 2, Narrative with clear evidence of an attachment script***
Tommy has just got a new **bike** and is out cycling for the first time. He bikes and bikes and bikes and suddenly his mom calls him that he has to hurry in before dinner is ready. When Tommy is about to ride home he cycles on a big rock and rolls over with his bike and **hurts** himself. All because he hurried… **Mom** she comes **hurrying** and bouncing because Tommy **he cries** so loud. Mom, she hugs him and tells him that they have to go to the **doctor** to get a tetanus **shot**. Tommy definitely does not want to, but then Mom says they can go buy a **toy** he has wanted for a long time, if he follows to the doctor and takes the shot. **Mom** holds him until his tears **stop**, and they go. When they come into the doctor they are greeted by a huge smile and the doctor says “hello” and Mom sits next to Tommy and **holds** his hand and then Tommy feels that there is no danger in getting the shot.

Note. Prompt-words are marked in bold. In Story 1, prompt words “mother", ”stop”, and “hold” were not used.

**Table 2:** Description of Study Participants (*N* = 37).

	*N*	(%)
Number of children		
=1	27	(72.9%)
=2	7	(18.9%)
≥3	3	(8.2%)
Sex of youngest (focus) child		
female	17	(46.0%)
male	20	(54.0%)
Marital Status		
Married	25	(67.5%)
Cohabiting	8	(21.6%)
Single/Divorced	4	(10.8%)
Education		
University or equivalent	31	(84.0%)
Upper secondary	6	(16.0%)
Current occupation		
Full-time employment	6	(16.2%)
Part-time employment	19	(51.3%)
Parental leave	5	(13.5%)
Student	3	(8.1%)
Unemployed	2	(5.4%)
Sick leave	2	(5.4%)

**Table 3:** Means (SD) for attachment (ASA) for the different attachment style questionnaire (ASQ) attachment orientations.

	ASQ			
	Secure (*n* = 15)	Avoidant (*n* = 10)	Anxious + Disorganized (*n* = 12)	*F*_(2, 36)_
ASA Parent–Child	4.06 (0.87)	2.98 (1.05)	3.07 (1.03)	5.02 *
ASA Adult–Adult	3.73 (1.10)	2.56 (0.59)	2.79 (1.00)	5.35 *
ASA Total	4.05 (0.76)	2.80 (0.71)	3.08 (0.91)	8.85 **

Note. ASA Total = mean score across all four stories; ASA Parent–Child = mean score for “Baby’s Morning” and “Doctor’s Office”; ASA Adult–Adult = mean score for “Accident” and “Camping Trip”. For statistical power in this analysis, ASQ Disorganized profiles were analyzed together with Anxious profiles, as the anxious components were strongest in both. All post-hoc comparisons (Scheffé) between Secure and Avoidant, and between Secure and Anxious + Disorganized were significant with *p* < 0.05. * *p* < 0.01, and ** *p* < 0.001.
